# Intelligence and executive functions in frontotemporal dementia

**DOI:** 10.1016/j.neuropsychologia.2013.01.008

**Published:** 2013-03

**Authors:** María Roca, Facundo Manes, Ezequiel Gleichgerrcht, Peter Watson, Agustín Ibáñez, Russell Thompson, Teresa Torralva, John Duncan

**Affiliations:** a Institute of Cognitive Neurology (INECO), Buenos Aires, Argentina; b Institute of Neurosciences, Favaloro University, Buenos Aires, Argentina; c Laboratory of Neuroscience, Universidad Diego Portales, Santiago, Chile; d MRC Cognition and Brain Sciences Unit, 15 Chaucer Road, Cambridge CB2 7EF, UK

**Keywords:** Frontotemporal dementia, Fluid intelligence, Executive functions, Theory of mind, Multitasking

## Abstract

Recently (Roca et al. (2010), we used the relationship with general intelligence (Spearman’s *g*) to define two sets of frontal lobe or “executive” tests. For one group, including Wisconsin card sorting and verbal fluency, reduction in *g* entirely explained the deficits found in frontal patients. For another group, including tests of social cognition and multitasking, frontal deficits remained even after correction for *g*. Preliminary evidence suggested a link of the latter tasks to more anterior frontal regions. Here we develop this distinction in the context of behavioural-variant frontotemporal dementia (bvFTD), a disorder which progressively affects frontal lobe cortices. In bvFTD, some executive tests, including tests of social cognition and multitasking, decline from the early stage of the disease, while others, including classical executive tests such as Wisconsin card sorting, verbal fluency or Trail Making Test part B, show deficits only later on. Here we show that, while deficits in the classical executive tests are entirely explained by *g*, deficits in the social cognition and multitasking tests are not. The results suggest a relatively selective cognitive deficit at mild stages of the disease, followed by more widespread cognitive decline well predicted by *g*.

## Highlights

► A progressive loss of general intelligence (*g*) can be found in patients with FTD. ► Advanced FTD classical executive deficits can be entirely explained by loss of *g*. ► Early social cognition and multitasking deficits exceed the ones predicted from *g*. ► Assessment in FTD should encompass *g*, multitasking and social cognition.

## 1 Introduction

The prefrontal cortex is a key element in the achievement of effective behaviour and higher cognitive function. To assess frontal lobe functions, clinical and experimental neuropsychology (Spearman, 1904) have successfully designed many tests of “executive” processing, including the Wisconsin card sorting tests (WCST), verbal fluency, trail making test B (TMTB), and many more. Quite commonly, however, patients with frontal pathology have been described as presenting marked cognitive and behavioural deficits yet performing within an average range on classical executive tests (Burgess, 2000; Eslinger & Damasio, 1985; Goldstein, Bernard, Fenwick, Burgess, McNeil, 1993; Metzler & Parkin, 2000; Shallice & Burgess, 1991). The results suggest a degree of dissociation among frontal lobe functions, only some of which are well captured in classical tests.

Recently (Woolgar et al., 2010), we developed this finding in a study of executive impairments and loss of “general intelligence” or Spearman’s *g*. Studies using functional brain imaging link conventional tests of *g* to activity in a specific network of frontal and parietal brain regions, including cortex along the inferior frontal sulcus, the anterior insula/frontal operculum, the dorsomedial frontal cortex including dorsal anterior cingulate and pre-supplementary motor area, and cortex along the intraparietal sulcus (Bishop, Fossell,a, Croucher, & Duncan, 2008; Duncan & Owen, 2000; Prabhakaran, Smith, Desmond, Glover, & Gabrieli, 1997). Damage within this same network predicts reduction in *g* (Woolgar et al., 2010). In our study, we showed that *g* was a substantial contributor to many frontal deficits. Particularly, for classical executive tasks, including the WCST and verbal fluency, deficits in frontal lobe patients were entirely explained by their loss of *g*. However, on a second set of frontal tasks, deficits remained even after *g* was statistically controlled. Included in this latter group were tests of theory of mind and multitasking. Tentatively, we linked deficits in this second set of tests to damage in anterior frontal cortex, in line with strong anterior activity in functional imaging studies (Gilbert et al., 2006; though note also involvement of anterior regions in classical tests of *g*, see Christoff et al., 2001; Gläscher et al., 2010).

Here, we develop our previous conclusions in the context of patients with the behavioural variant of frontotemporal dementia (bvFTD), a degenerative disorder whose clinical manifestations include changes in personality, impaired social interaction, disinhibition, deficits in impulse control and loss of insight (Hodges & Miller, 2001). Critically, bvFTD shows early involvement of medial orbitofrontal regions, extending to the frontal pole (Broe et al., 2003; Hornberger et al., 2010; Kipps, Nestor, Fryer, & Hodges, 2007; Rosen et al., 2002; Seeley, 2009, 2008), and in part separate from the more dorsal and lateral network that is supposedly linked to *g*. From a neuropsychological perspective, in early stages of the disease, tests of social cognition and multitasking have shown a greater sensitivity in the detection of bvFTD than classical executive tests (Gleichgerrcht, Ibáñez, Roca, Torralva, & Manes, 2010; Rahman, Sahakian, Hodges, Rogers, & Robbins, 1999; Torralva, Roca, Gleichgerrcht, Bekinschtein, & Manes, 2009; Torralva et al., 2007). Only as the disease progresses do classical executive tests, such as the WCST and verbal fluency, begin to decline (Miller et al., 1991; Neary, Snowden, Northen, & Goulding, 1988; Torralva et al., 2009), possibly reflecting the more advanced involvement of additional prefrontal areas (Williams, Nestor, & Hodges, 2005). In a recent study (Torralva et al., 2009), for example, we assessed a group of bvFTD with classical executive tests and with an Executive and Social Cognition Battery, which included theory of mind tests (mind in the eyes, faux pas), multitasking (hotel task) and decision making (Iowa gambling task). Patients were divided into two groups according to their general cognitive performance. Low functioning patients – presumably reflecting a more advanced state of the disease – differed from controls on classical executive tasks and the Executive and Social Cognition Battery. In contrast, only the latter showed deficits in high functioning patients.

Here, we tested the hypothesis that links to *g* help explain the distinction between classical executive tests and those tests which have shown greater sensitivity in the detection of early bvFTD, including multitasking and social cognition. For these early-declining tests, we predicted, deficits arise in frontal networks not closely related to *g*, and accordingly should remain even once *g* is statistically controlled. In contrast, for the classical executive tests, likely impaired only in more advanced patients, deficits might be entirely explained by *g*. To test this hypothesis, we re-analysed our previous data set on patients with bvFTD (*n*=35) and control subjects (*n*=14). Conventionally, *g* can be measured either using a standard psychometric test such as Raven’s Matrices (Raven, Court, & Raven, 1988) or simply by averaging performance on a diverse battery of tasks; in practice, these two approaches give largely similar results (Cattell, 1971), and we used the latter method here. We thus asked which deficits in frontal tests remain, after correction for *g* as measured in a general test battery (GTB).

## 2 Material and methods

### 2.1 Subjects

Patients with a diagnosis of bvFTD (*n*=35) according to the Lund and Manchester criteria (Neary et al., 1998) were recruited as part of a broader ongoing study on frontotemporal dementia. All patients gave informed consent prior to inclusion and underwent a standard examination battery including neurological, neuropsychiatric and neuropsychological examinations. Patients were followed through time; all showed frontal atrophy on neuroimaging and did not meet criteria for specific psychiatric disorders, thus avoiding the inclusion of non-progressors or so-called ‘phenocopy’ cases in the analysed sample (Kipps et al., 2007; Manes, 2012). When retrospectively analyzed, all of the patients included in the study met the revised criteria for probable bvFTD (Rascovsky et al., 2011).

Based on previous reports (Torralva et al., 2009), bvFTD participants were further subdivided according to their cognitive performance. A patient was included in the high functioning FTD (hf-FTD) group when he/she showed a score above 86/100 (the standard cut-off for dementia) in the ACE (Mathuranath, Nestor, Berrios, Rakowicz, & Hodges, 2000), a screening tool able to detect progression of disease in FTD (Kipps, Nestor, Dawson, Mitchell, & Hodges, 2008) and which has been demonstrated to correlate with the degree of atrophy found in the disease (Kipps et al., 2007). When a patient showed a score below the cut-off in such test, he/she was included in the low functioning FTD (lfFTD) group. This procedure resulted in 16 participants with ACE scores above the cut-off (hfFTD) and a group of 19 participants with ACE scores below cut-off (lfFTD).The mean age of hfFTD patients was 65.0 years (SD=7.4) and mean years of education 13.8 (SD=3.8). The mean age of lfFTD patients was 69.1 years (SD=5.7) and mean years of education 13.5 (SD=5.2).

Healthy control subjects (*n*=14) were recruited from the same geographical area as the patients and were matched for age and level of education. The mean age of controls was 65.5 years (SD=6.5) and mean years of education 13.9 (SD=3.1).

### 2.2 Neuropsychological assessment

#### 2.2.1 Wisconsin card sorting test (Nelson, 1976)

For the Wisconsin Card Sorting Test we used Nelson’s modified version of the standard procedure. Cards varying on three basic features – colour, shape and number of items – must be sorted according to each feature in turn. The participant’s first sorting choice becomes the correct feature, and once a criterion of six consecutive correct sorts is achieved, the subject is told that the rules have changed, and cards must be sorted according to a new feature. After all three features have been used as sorting criteria, subjects must cycle through them again in the same order as they did before. Each time the feature is changed, the next must be discovered by trial and error. Score was total number of errors, either before successful completion of all six task stages, or after a maximum of 48 cards.

#### 2.2.2 Verbal fluency (Benton & Hamsher, 1976)

In verbal fluency tasks, the subject generates as many items as possible from a given category. We used the standard phonemic version, asking subjects to generate words beginning with the letters F, A and S in successive blocks of 1 min/letter. Score was the total number of correct words generated.

#### 2.2.3 Trail making test B (Partington & Leiter, 1949)

The trail making test consists of two parts. In the present study part B was administered (TMTB). In this test the subject is required to draw lines sequentially connecting 13 numbers and 12 letters distributed on a sheet of paper. Letters and numbers are encircled and must be connected alternatively (e.g., 1, A, 2, B, 3, C, etc.). Score was the total time (s) required to complete the task, given a negative sign so that high scores meant better performance.

#### 2.2.4 Hotel task (Manly, Hawkins, Evans, Woldt, & Robertson, 2002; Torralva et al., 2009)

The task comprised five primary activities related to running a hotel (compiling bills, sorting coins for a charity collection, looking up telephone numbers, sorting conference labels, proofreading). The materials needed to perform these activities were arranged on a desk, along with a clock that could be consulted by removing and then replacing a cover. Subjects were told to try at least some of all five activities during a 15 min period, so that, at the end of this period, they would be able to give an estimate of how long each task would take to complete. It was explained that time was not available to actually complete the tasks; the goal instead was to ensure that every task was sampled. Subjects were also asked to remember to open and close the hotel garage doors at specified times (open at 6 min, close at 12 min), using an electronic button. Of the several scores possible for this task, we used time allocation: for each primary task we assumed an optimal allocation of 3 min, and measured the summed total deviation (in seconds) from this optimum. Total deviation was given a negative sign so that high scores meant better performance.

#### 2.2.5 Iowa gambling task (Bechara, Damasio, & Damasio, 2000)

In the Iowa gambling task, subjects are required to pick cards from four decks and receive rewards and punishments (winning and losing abstract money) depending on the deck chosen. Two ‘risky’ decks yield greater immediate wins but very significant occasional losses. The other two ‘conservative’ decks yield smaller wins but negligible losses that result in net profit over time. Subjects make a series of selections from these four available options, from a starting point of complete uncertainty. Reward and punishment information acquired on a trial by trial basis must be used to guide behaviour towards a financially successful strategy. Normal subjects increasingly choose conservative decks over the 100 trials of the task. Our score was the total number of conservative minus risky choices. Data were available for all patients and 10 control subjects.

#### 2.2.6 Faux pas (Stone, Baron-Cohen, & Knight, 1998)

In each trial of this test, the subject was read a short, one paragraph story. To reduce working memory load, a written version of the story was also placed in front of the subject. In 10 stories there was a faux pas, involving one person unintentionally saying something hurtful or insulting to another. In the remaining 10 stories there were no faux pas. After each story, the subject was asked whether something inappropriate was said and if so, why it was inappropriate. If the answer was incorrect, an additional memory question was asked to check that basic facts of the story were retained; if they were not, the story was re-examined and all questions repeated. The score was 1 point for each faux pas correctly identified, or non-faux pas correctly rejected.

#### 2.2.7 Mind in the eyes (Baron-Cohen, Jolliffe, Mortimore, & Robertson, 1997)

This task consisted of 17 photographs of the eye region of different human faces. Participants were required to make a two alternative forced choices that best described what the individual was thinking or feeling (e.g., worried-calm). The score was total number correct.

#### 2.2.8 General test battery (GTB)

All participants were also assessed with a general test battery used to derive a measure of *g*. This battery included Forward Digit Span task (Wechsler, 1997a), Letters and Numbers (Wechsler, 1997a), logical memory test (Wechsler, 1997b), Rey auditory verbal learning test (Rey, 1941), Rey complex figure test (Rey, 1941), and Raven’s coloured progressive matrices (Raven, 1996). For this set of tests, principal component analysis produced a first component accounting for 62% of the total variance. Loadings on this component were moderate to high for all tests (range=0.58–0.86). The *g* score for each participant (gGTB) was defined as the score on this first principal component.

#### 2.2.9 Language proficiency

Given that most tasks used in this study relied on language abilities, which are known to be affected in bvFTD, all subjects were also assessed with tests of language proficiency. Language assessment comprised tests of naming (a 20 item version of the Boston naming test) (Kaplan, Goodglass, & Weintraub, 1983) and semantic knowledge (pyramids and palm trees test) (Howard & Patterson, 1992).

## 3 Results

**Fig. 1:**
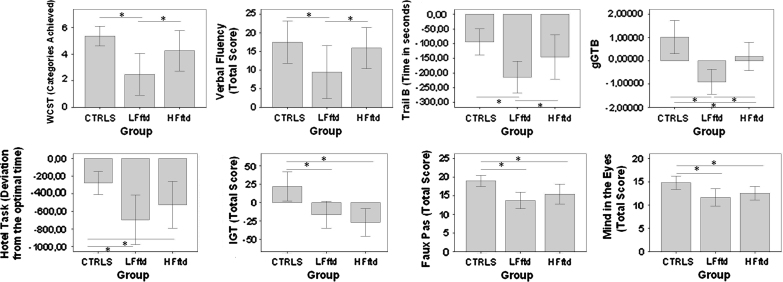
Group performance on each task. Significant differences are indicated by asterisks (*p*<0.05).

Groups were well matched for age, *F*(2,46)=1.34, *P*=0.18, and years of education, *F*(2,46)=0.53, *P*=0.95 (Table 1). For executive tests and gGTB, the mean scores of each group are shown in Fig. 1. For each executive test, groups were compared by analysis of variance (ANOVA) with post hoc comparisons. Results are summarized in Table 2 (see also Torralva et al., 2009). Executive tests showed two different profiles of impairment. For the classical executive tasks, WCST, verbal fluency, and TMTB, a mild and non-significant impairment in hfFTD patients became a substantial deficit in the lfFTD group. For remaining tasks – faux pas, mind in the eyes, hotel task and IGT – deficits were already substantial in the hfFTD group, and only slightly more marked for lfFTD.

For gGTB, ANOVA also showed significant differences between the 3 groups, *F*(2,46)=40.7, *P*<0.01. On this measure, resembling results for the classical executive tasks, hfFTD patients were somewhat closer to controls than to lfFTD patients (Fig. 1), though post hoc comparisons showed significant differences between all groups (hfFTD vs. controls, *P*<0.01; hfFTd vs. lfFTD, *P*<0.01; lfFTD vs. controls, *P*<0.01).

**Fig. 2:**
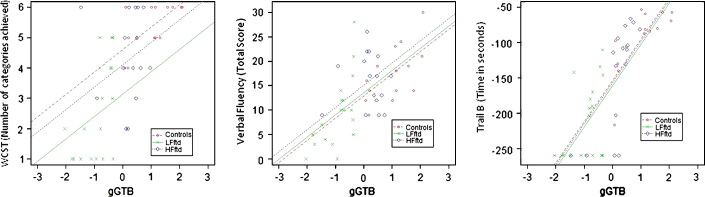
Scatter plots relating gGTB to the three classical frontal tests. Regression lines reflect the average within-group association of the two variables, as determined by ANCOVA, constrained to have the same slope across groups.

Scatter plots relating gGTB to the three classical frontal tests are shown in Fig. 2. For all classical executive tests, scores were heavily dependent on gGTB, and once this influence was removed by analysis of covariance (ANCOVA), group differences were no longer significant (Table 2). Regression lines in Fig. 2 come from the standard ANCOVA model, reflecting the average within-group association of the two variables and constrained to have the same slope across groups. As calculated from the corresponding variance terms of the ANCOVA, average within-group correlations with gGTB were 0.33 for WCST, 0.45 for verbal fluency, and 0.61 for TMTB.

**Fig. 3:**
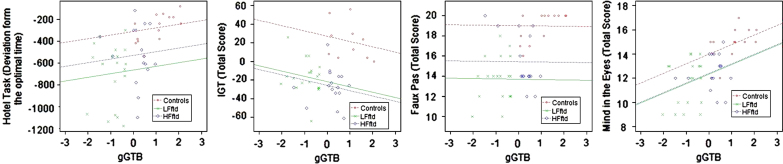
Scatter plots relating gGTB to the social cognition and multitasking tests. Regression lines reflect the average within-group association of the two variables, as determined by ANCOVA, constrained to have the same slope across groups.

Scatter plots relating gGTB to the other frontal tests are shown in Fig. 3. For these tests results were very different. Except for Mind in the Eyes, scores were barely related to gGTB, with average within-group correlations of 0.08 for the Hotel Task, 0.18 for Iowa Gambling Task, 0.01 for Faux Pas, and 0.29 for Mind in the Eyes. Using ANCOVA to remove the influence of gGTB left significant group differences for 3 of the 4 tests, with the exception being a value of *P*=0.06 for mind in the eyes (Table 2).

As expected, groups also differed significantly on tests of language proficiency (Table 1), both on the Boston naming test, *F*(2,46)=7.12, *P*<0.01, and in the pyramids and palm trees test, *F*(2,46)=4.51, *P*▒=▒0.01. Within the patient group, no significant correlations were found between performance on executive and language tests.

## 4 Discussion

In the present study we aimed to investigate the relationship between frontal deficits and *g* in bvFTD. We derived a *g* score (gGTB) for 35 bvFTD patients and 14 control subjects, all of whom were also assessed with a variety of frontal tasks previously reported as impaired in different stages of the disease. As expected by the established progression of neuropathological changes in bvFTD, classical executive tests showed a different relationship with *g* than social cognition and multitasking tests sensitive to early bvFTD. For three of the latter - Hotel Task, Iowa Gambling Task, and Faux Pas -performance was only weakly related to *g*, and even removing any influence of *g*, significant differences remained between patients and controls. A similar trend was seen for the fourth test in this group, mind in the eyes. For classical executive tests, the link to *g* was stronger, and group differences were no longer significant once the effect of *g* was removed. In general, the performance of bvFTD patients on executive tests was not significantly related to language deficits.

Our findings are largely compatible with those we previously obtained in patients with focal frontal lesions (Roca et al., 2010). In focal patients too, deficits in classical executive tests (WCST, verbal fluency) were entirely explained by reduction in *g*, while deficits in multitasking and social cognition were not. While in focal patients we presented data only on the WCST and verbal fluency, in the present study we also demonstrated that the TMTB behaves similarly to those classical executive tests: once *g* is introduced as a covariate, differences between groups are no longer present.

An important difference between FTD and focal patients, however, concerns the Iowa Gambling Task. In a previous study we found that in patients with focal lesions, deficits on the IGT were no longer present once fluid intelligence was included as a covariate, while in the present patients, deficits in this task far exceeded predictions from *g* scores. These results are compatible with previous studies showing prominent decision making deficits in FTD (Gleichgerrcht et al., 2010; Manes et al., 2011; Rahman et al., 1999; Torralva et al., 2009, 2007). While the Iowa gambling task has been conventionally linked to ventromedial frontal functions (Bechara, Damasio, Damasio, & Anderson, 1994) further studies revealed that damage to other regions within the frontal cortex could also affect performance on this task (Bechara, Damasio, Tranel, & Anderson, 1998; MacPherson, Phillips, Della Sala, & Cantagallo, 2009; Manes et al., 2002). Such results suggest a multicomponent test, and in this light, the different results for focal and bvFTD patients may likely be traced to their very different lesion characteristics. In our previous focal patients, there were few cases of ventromedial damage; in this group, impairments may be dominated by the general cognitive requirements of the Iowa Gambling Task, and thus closely related to *g*. In bvFTD, in contrast, the picture may be dominated by ventromedial frontal pathology, and by specific deficits in risky decision-making.

For clinical assessment of bvFTD, our data highlight the necessity of including frontal functions that are not routinely considered yet are crucial to competent everyday life performance, such as contextual social cognition, multitasking and mentalizing (Burgess, Alderman, Volle, Benoit, & Gilbert, 2009; Torralva et al., 2009). Moreover, the early fronto-insular-temporal atrophy pattern of bvFTD (Seeley, 2008; Viskontas, Possin, & Miller, 2007; Williams et al., 2005) seems to be specifically associated with contextual integration processes recruited during situated social and ecological cognition tasks (Ibáñez & Manes, 2012). Thus, in the early phase, bvFTD would be a specific disorder of contextual integration in social cognition domains (Ibáñez & Manes, 2012). Our data, consistent with this hypothesis, showed impairments in multitasking, decision making and theory of mind to be a core domain of bvFTD, suggesting an early involvement of brain networks engaged in situated-contextual cognition. This core set of deficits seem to be extended by later atrophy of a more dorsal and lateral frontal network, associated with a general cognitive decline and reduction in *g*.

Undoubtedly, the rather general cognitive functions reflected in *g* will influence success in most or all cognitive tests. Removing this influence, we suggest, may clarify the more specific impairments that neuropsychological tests are generally intended to measure. Here, we have shown that this procedure helps clarify the progression of executive impairment in bvFTD. Specifically, it distinguishes a group of early, relatively focal cognitive impairments from a later, more global cognitive decline.

## Figures and Tables

**Table 1:** Demographical, cognitive status and language proficiency data for controls, lfFTD and hfFTD groups.

Variables	Controls (*n*=14)	lfFTD (*n*=19)	hfFTD (*n*=16)	Controls vs. lfFTD	Controls vs. hfFTD	hfFTD vs. lfFTD
Demographical data						
Age	65.5 (6.5)	69.1 (5.7)	65.0 (7.4)	>0.1	>0.1	>0.1
Education (years)	13.9 (3.0)	13.5 (5.2)	13.8 (3.8)	>0.1	>0.1	>0.1
Cognitive status						
MMSE	29.2 (1.0)	25.7 (3.2)	28.2 (1.9)	**<0.01**	>0.1	**<0.01**
ACE	94.5 (5.3)	74.2 (8.4)	91.0 (2.6)	**<0.01**	>0.1	**<0.01**
Language proficiency						
Boston naming test	19.8 (0.4)	16.8 (3.6)	18.9 (1.2)	**>0.01**	>0.1	**0.03**
Pyramids and palm trees test	51.8 (0.3)	48.7 (3.8)	50.5 (2.8)	**0.02**	0.74	0.22

Values are shown as mean (SD). ACE=Addenbrooke’s cognitive examination; MMSE=mini-mental state examination.

**Table 2:** Significance of group differences before and after gGTB was introduced as a covariate.

Variables	Main effect of group before gGTB was introduce as a covariate^a^ (*df*=2, 46^b^)	Controls vs. s lfFTD	Controls vs. hfFTD	hfFTD vs. lfFTD	Main effect of group after gGTB was introduced as a covariate^c^	Controls vs. lfFTD	Controls vs. hfFTD	hfFTD vs. lfFTD
WCST	*F*=18.6, ***P*****<0.01**	**< 0.01**	0.09	**< 0.01**	>0.1	>0.1	>0.1	>0.1
Verbal fluency	*F*=8.1, ***P*****<0.01**	**< 0.01**	>0.1	**0.01**	>0.1	>0.1	>0.1	>0.1
TMTB	*F*=16.9, ***P*****<0.01**	**< 0.01**	0.07	**< 0.01**	>0.1	>0.1	>0.1	>0.1
Hotel Task	*F*=12.0, ***P*****<0.01**	**< 0.01**	**0.02**	>0.1	**0.04**	**0.04**	>0.1	>0.1
Iowa gambling task	*F*=21.7, ***P*****<0.01**	**< 0.01**	**<0.01**	>0.1	**<0.01**	**<0.01**	**<0.01**	>0.1
Faux pas	*F*=23.5, ***P*****<0.01**	**< 0.01**	**< 0.01**	0.08	**<0.01**	**<0.01**	**<0.01**	>0.1
Mind in the eyes	*F*=15.6, ***P*****<0.01**	**< 0.01**	**< 0.01**	>0.1	0.06	>0.1	0.06	>0.1

a To compare performance between the groups a one-way ANOVA design with Bonferroni’s post hoc comparisons was used.

b Except for the Iowa gambling task (*df*=2, 42).

c To compare performance between the groups a one-way ANCOVA design with Bonferroni’s post hoc comparisons was used.
